# Enhancing Cu^2+^ Ion Removal: An Innovative Approach Utilizing Modified Frankincense Gum Combined with Multiwalled Carbon Tubes and Iron Oxide Nanoparticles as Adsorbent

**DOI:** 10.3390/molecules28114494

**Published:** 2023-06-01

**Authors:** Mushtaq Hussain, Syed Sulaiman Hussaini, Mohammad Shariq, Hanan Alzahrani, Arafa A. Alholaisi, Samar H. Alharbi, Sirajah A. Alsharif, Wafa Al-Gethami, Syed Kashif Ali, Abdel-Nasser M. A. Alaghaz, Mohd Asim Siddiqui, Kondaiah Seku

**Affiliations:** 1 Engineering Department, College of Engineering and Technology, University of Technology and Applied Sciences, Shinas 324, Oman; 2 Department of Physics, College of Science, Jazan University, Jazan 45142, Saudi Arabia; 3 Department of Physics, Al-Qunfudah University College, Umm Al-Qura University, Makkah 24382, Saudi Arabia; 4 Chemistry Department, Faculty of Science, Taif University, Al-Hawiah, Taif City P.O. Box 11099, Saudi Arabia; 5 Department of Chemistry, College of Science, Jazan University, Jazan 45142, Saudi Arabia

**Keywords:** multiwalled carbon, Cu adsorption, frankincense, adsorption kinetics, adsorption isotherm

## Abstract

Aquatic pollution, which includes organic debris and heavy metals, is a severe issue for living things. Copper pollution is hazardous to people, and there is a need to develop effective methods for eliminating it from the environment. To address this issue, a novel adsorbent composed of frankincense-modified multi-walled carbon nanotubes (Fr-MMWCNTs) and Fe_3_O_4_ [Fr-MWCNT-Fe_3_O_4_] was created and subjected to characterization. Batch adsorption tests showed that Fr-MWCNT-Fe_3_O_4_ had a maximum adsorption capacity of 250 mg/g at 308 K and could efficiently remove Cu^2+^ ions over a pH range of 6 to 8. The adsorption process followed the pseudo-second-order and Langmuir models, and its thermodynamics were identified as endothermic. Functional groups on the surface of modified MWCNTs improved their adsorption capacity, and a rise in temperature increased the adsorption efficiency. These results highlight the Fr-MWCNT-Fe_3_O_4_ composites’ potential as an efficient adsorbent for removing Cu^2+^ ions from untreated natural water sources.

## 1. Introduction

The addition of diverse pollutants, including heavy metals and organic matter, has changed the water’s natural composition. Eliminating heavy metals from water using adsorbents is an ongoing study area for fundamental research and practical applications [1,2]. The scientific and engineering communities face challenges regarding the expense, specificity, and efficient separation of these adsorbents from water [3,4]. Among these heavy metal ions, the copper ion (Cu^2+^) is particularly problematic during the electroplating process since it is more likely to leak into the water supply as a pollutant. Excessive exposure to copper ions can result in the development of chromosomal recessive [5]. The World Health Organization recommends a maximum of 2.0 mg/L copper concentration in drinking water, establishing the level at which copper should be controlled [6]. Essential techniques like chemical precipitation, electrochemistry, membrane technology, adsorption, and chemical reduction technology have been applied to eliminate Cu (II) ions from wastewater [7,8]. The targeted method depends on the purpose, operational cost, and treatment criteria. Adsorption techniques have seen the most utilization out of all methods over the past few years due to various advantages [9]. It is easy to use, very effective, inexpensive, and positively affects the environment. The adsorbents are capable of regenerating themselves, and this method produces no additional by-products [10]. Activated carbon with a size of a few nanometers, graphene oxide, and carbon nanotubes (CNTs) are just a few examples of the various forms of carbonaceous materials that have so far been manipulated as raw materials to create various adsorbents [11]. CNTs are a potential candidate among these materials because they have good electrical, chemical, and mechanical properties. Because of their excellent electrical, chemical, and mechanical characteristics, CNTs are the most suitable adsorbent materials [12,13]. There are two significant advantages to these materials. Firstly, they possess a higher surface area than other materials. Second, they can absorb various contaminants via electronic hydrophobic interactions because of the hexagonal arrangement of the atoms on their surface [14,15]. Numerous studies have highlighted the immense capacity of CNTs to adsorb various organic/inorganic pollutants [16,17]. CNTs are made of sp^2^-hybridized carbon atoms that exhibit unique properties, are highly polarizable, and have little ability to hold water. The separation of carbon nanotubes from solutions continues to be challenging [18]. 

Magnetic separation is a highly efficient physical separation method that is beneficial for recycling small nanoparticles in water purification processes. This method is characterized by its ability to achieve high separation efficiency using minimal energy [19]. Moreover, magnetic separation offers the additional benefit of preventing the aggregation of nanoparticles in the solution, as the added magnetism helps to keep the particles dispersed and separate from each other. Magnetic separation is a quick, easy, and cheap way to separate magnetic adsorbents from treated media after they have taken up pollutants. It is faster and more affordable than filtration and centrifugation.

Recently, metal oxide/CNTs nanocomposites have been the subject of extensive research and have proven to be highly useful adsorbents for removing heavy metals from wastewater [20]. Rahmani et al. used co-precipitation to make a magnetic nanocomposite of MnFe_2_O_4_ and bentonite that can adsorb Co(II) ions from wastewater. The nanocomposite proved to be very good at adsorbing Co(II) ions, which suggests that it could be an effective way to get heavy metals out of polluted water [21]. Chen et al. developed Fe_3_O_4_/CNTs magnetic nanoparticles that effectively eliminated Cr(VI) from wastewater. The nanoparticles demonstrated excellent magnetic separation efficiency at low magnetic field gradients [22]. Wang and colleagues developed a hybrid adsorbent composed of γ-PGA-Fe_2_O_4_-GO-(o-MWCNTs) nanomaterials, demonstrating high efficiency in the adsorption of metal ions from contaminated water [23]. Yin and colleagues developed a multifunctional adsorbent of metal–organic frameworks (MOFs), UiO-66, decorated with Fe_2_O_3_ magnetic nanoparticles, termed UiO-66@Fe_2_O_3_@UiO-66, for the efficient elimination of cosmetics and pharmaceuticals from water [24]. A magnetic CoFe-LDH/g-C_3_N_4_ nanocomposite reported by Ou et al. successfully removed the Cr(Ⅵ) from aqueous solution [25].

Due to their effectiveness, specificity, reusability, and affordability, metal oxide nanoparticles are a viable option for the removal of heavy metal ions [26]. Many research projects have successfully removed metal ions via nanoadsorbents made of metal oxides such as Fe_2_O_3_, ZnO, TiO_2_, etc. [27,28,29]. Fe_3_O_4_ nanoparticles are highly appealing for modifying CNTs due to their distinct magnetic properties compared to other metal oxide nanoparticles. With nanoadsorbent technology, pollution-related problems can be solved affordably and sustainably [30]. This technology can effectively remove or minimize various pollutants, including organic and inorganic contaminants, while avoiding the generation of harmful by-products and toxic intermediates. Their toxicity and pollutant removal time are disadvantages [29,31]. Modifying capping agents with active groups can enhance adsorption efficiency [32,33]. The toxicity problem can be solved by using organic plant materials as stabilizing and capping agents and techniques for making nanoadsorbents that are safe for the environment [34,35]. We previously described the synthesis of Au and Pd nanoparticles from frankincense resin and the efficiency of these particles in catalyzing dye degradation [36,37]. 

The current investigation suggests a sustainable approach to synthesizing modified frankincense resin gum-multiwalled carbon nanotube-iron oxide nanoadsorbents (Fr-MWCNT-Fe_3_O_4_). Various techniques were used to characterize the synthesized materials, including UV/visible spectroscopy, TEM, SEM, FTIR, XRD, DLS, and zeta potential. The primary goal of this study is to assess the effectiveness of these nanoadsorbents in eliminating Cu^2+^ ions from industrial wastewater. The study systematically examined various adsorption models, including adsorption isotherms, kinetics, and thermodynamics models. The nanomaterial exhibited exceptional magnetic separation and adsorption capabilities. Moreover, the potential environmental applications of this adsorbent were assessed in the study.

## 2. Results and Discussion

### 2.1. Characterization of Fr-MMWCNT and Fr-MMWCNT-Fe_3_O_4_

**Figure 1:**
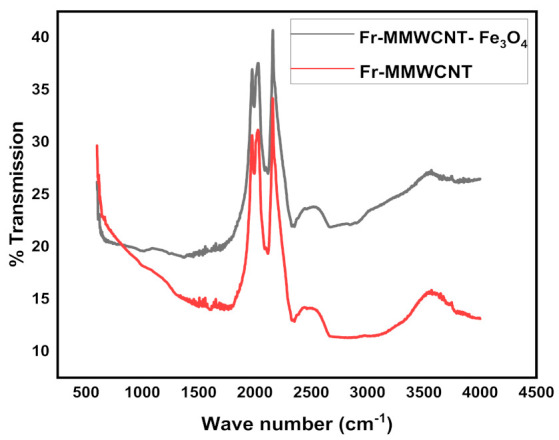
FT-IR spectra of Fr-MMWCNT and Fr-MMWCNT-Fe_3_O_4_.

The peaks observed in the frankincense-based multiwalled carbon nanotube (Fr-MMWCNT) FTIR spectrum (Figure 1) at 2330.6 cm^−1^ correspond to the -C–O bonds, 2115.8 cm^−1^ and 2088.9 cm^−1^ correspond to the carbon–carbon triple bond (C≡C) in nitrile and isocyanide, 1990.3 cm^−1^ to (C=C) in the aromatic ring of the frankincense-based compound, and 1834.1 cm^−1^ to the carbonyl group (C=O) in the ester functional groups, respectively [38,39,40,41,42]. The FTIR peaks observed in the multiwalled carbon with iron oxide composite at 2117.59 cm^−1^, 1995.73 cm^−1^, 2329 cm^−1^, 2089 cm^−1^, and 614 cm^−1^ correspond to various stretching vibrations (Figure 1). The transmittance peak observed at 2117.59 cm^−1^ designates the presence of C–C stretching vibrations in the carbon nanotubes. The peak detected at 1995.73 cm^−1^ is attributed to C–O stretching vibrations, indicating the presence of oxygen-containing functional groups on the carbon nanotube surface [43]. The peak observed at 2089 cm^−1^ is attributed to C=C stretching vibrations, representing the carbon-carbon double bonds in the carbon nanotubes. Finally, the peak observed at 614 cm^−1^ is due to the Fe–O stretching vibrations of the iron oxide nanoparticles, indicating the existence of iron oxide on the carbon nanotube surface [44].

**Figure 2:**
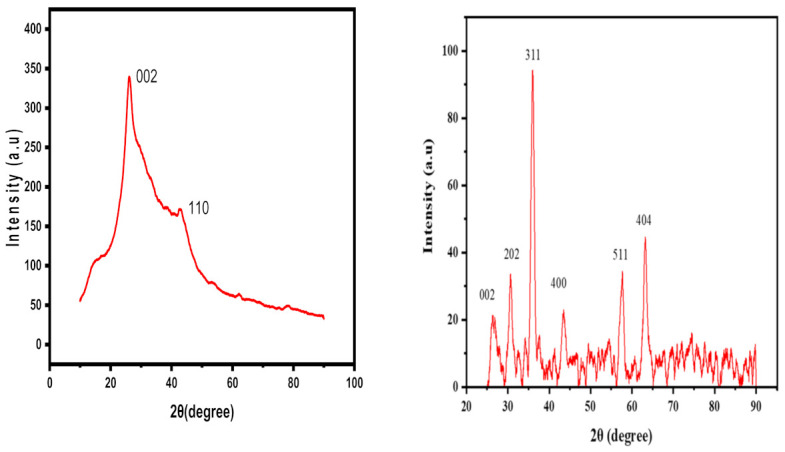
XRD peaks for Fr-MMWCNT and Fr-MMWCNT-Fe_3_O_4_.

In the X-ray diffraction pattern of the Fr-MMWCNT, the peaks observed at 2θ values of 26.24° and 42.5° correspond to the (002) and (110) planes of the MWCNTs structure [45,46]. In the pattern of the Fr-MMWCNT-Fe_3_O_4_ (Figure 2), the peaks observed at 2θ values of 26.24° and 42.5° correspond to the (002) and (110) planes of the MWCNTs structure, respectively (Figure S1. JCPDS No. 01-1061) [47]. The XRD pattern of Fr-MMWCNT-Fe_3_O_4_ reveals weak diffraction peaks for MWCNTs, indicating that the CNT’s structure is preserved in the composite material [11]. The diffraction peaks observed at 2θ values of 30.65°, 35.98°, 43.49°, 57.56°, and 63.22° are attributed to the presence of iron oxide nanoparticles (Figure S2. JCPDS No. 00-2321) in the corresponding (202), (311), (400), (511), and (404) planes of the Fe_3_O_4_ structure. This confirms the presence of the magnetic structure of the Fe_3_O_4_ nanoparticles in the composite without any impurities [12].

**Figure 3:**
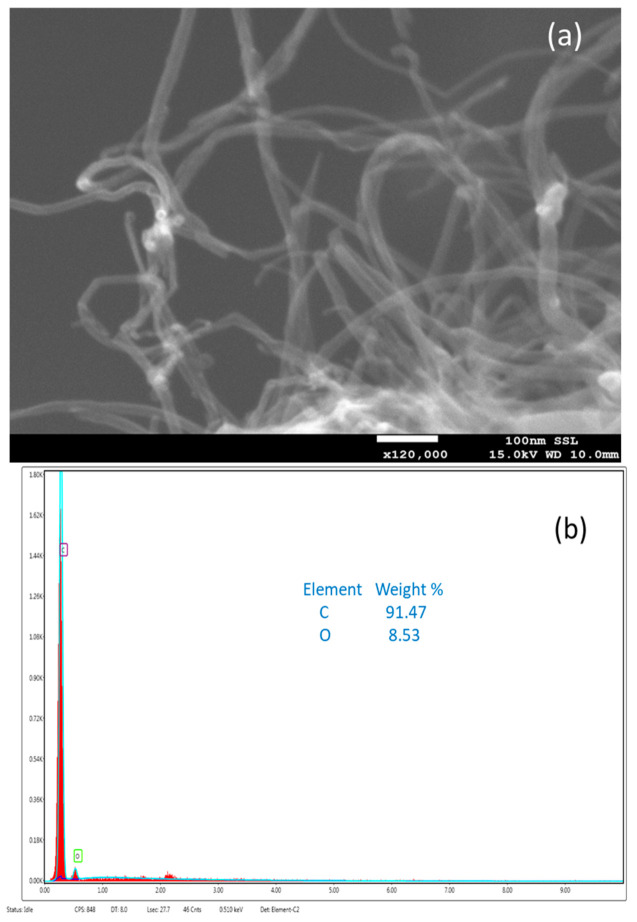
SEM (**a**) and EDS (**b**) of Fr-MMWCNT.

**Figure 4:**
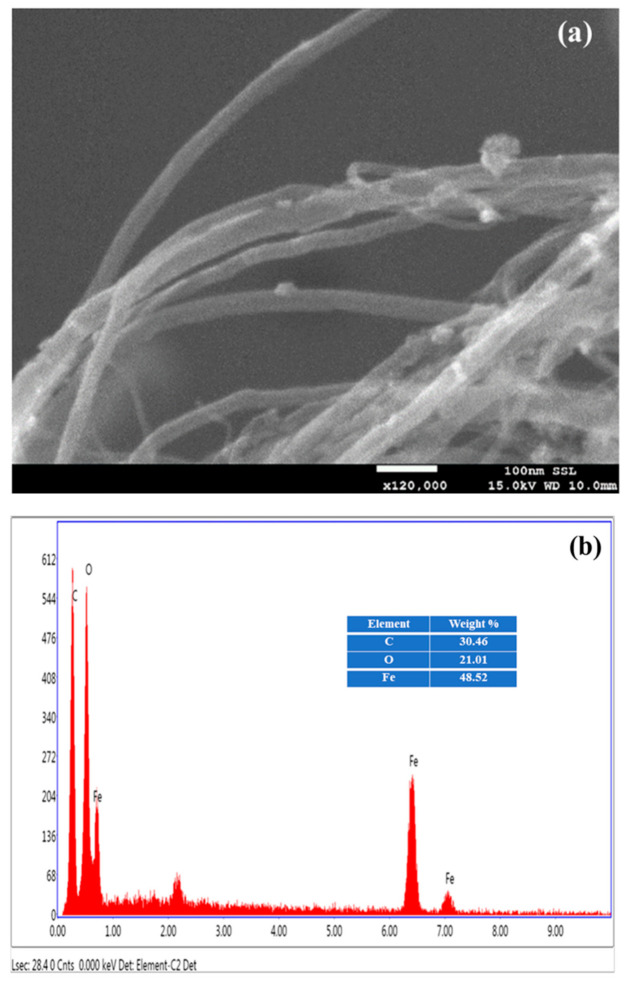
SEM (**a**) and EDS (**b**) of Fr-MMWCNT-Fe_3_O_4_ composite.

The Fr-MMWCNT composite’s scanning electron microscopy (SEM) image indicated a coating of Fr-gum on the nanotubes, as seen in the SEM image (Figure 3a). According to EDS analysis of the samples, carbon and oxygen were found in a ratio of 91.47% to 8.53%. The scanning electron microscope (SEM) image of the Fr-MMWCNT-Fe_3_O_4_ composite revealed that the nanotube surfaces were covered with a layer of Fr-gum, indicating the successful preparation of the composite (Figure 4a). Additionally, the SEM image showed the presence of randomly distributed magnetite nanoparticles on the surface of the composite. The distribution of magnetite particles appeared to be clustered, meaning that the particles were not evenly distributed across the surface. The clustering of magnetite particles could be due to several reasons. 

Overall, the SEM image of the Fr-MMWCNT-Fe_3_O_4_ composite provides valuable insight into the morphology and structure of the composite material, showing the presence of Fr-gum on the nanotube surface and the distribution of magnetite particles in clusters. 

The EDS analysis has detected the presence of carbon, oxygen, and iron in the ratio of 30.46% carbon, 21.01% oxygen, and 48.52% iron in the Fr-MMWCNT-Fe_3_O_4_ composite (Figure 4b).

**Figure 5:**
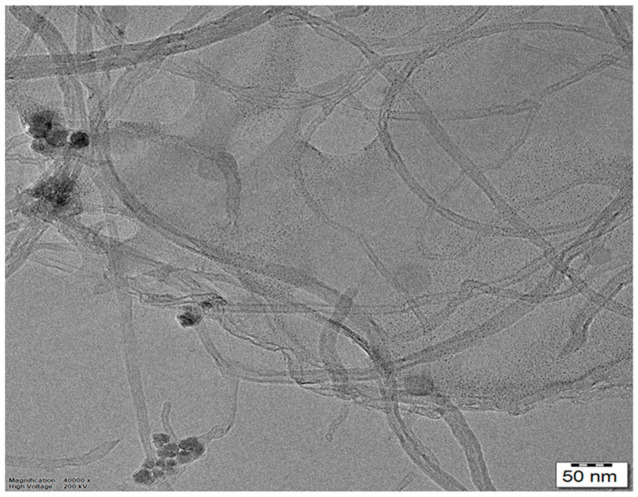
HRTEM of Fr-MMWCNT-Fe_3_O_4_ composite.

The HRTEM evaluation of the Fr-MMWCNT-Fe_3_O_4_ composite discloses a carbon nanotube with a cylindrical shape enclosed by gum material and iron oxide nanoparticles around it (Figure 5). These particles are randomly dispersed over the carbon nanotube. The distance between the two carbon layers is measured to be 15–60 nm, while the diameter of the nanotube is approximately 9–13 nm. The iron oxide nanoparticles display assorted sizes, ranging from 10–25 nm. The carbon nanotubes are several micrometers in length and exhibit smooth surfaces and substantial specific surface areas. The diffraction rings observed in the SAED image verify the magnetite nanoparticles’ crystalline nature in the composite.

### 2.2. Adsorption Parameters 

**Figure 6:**
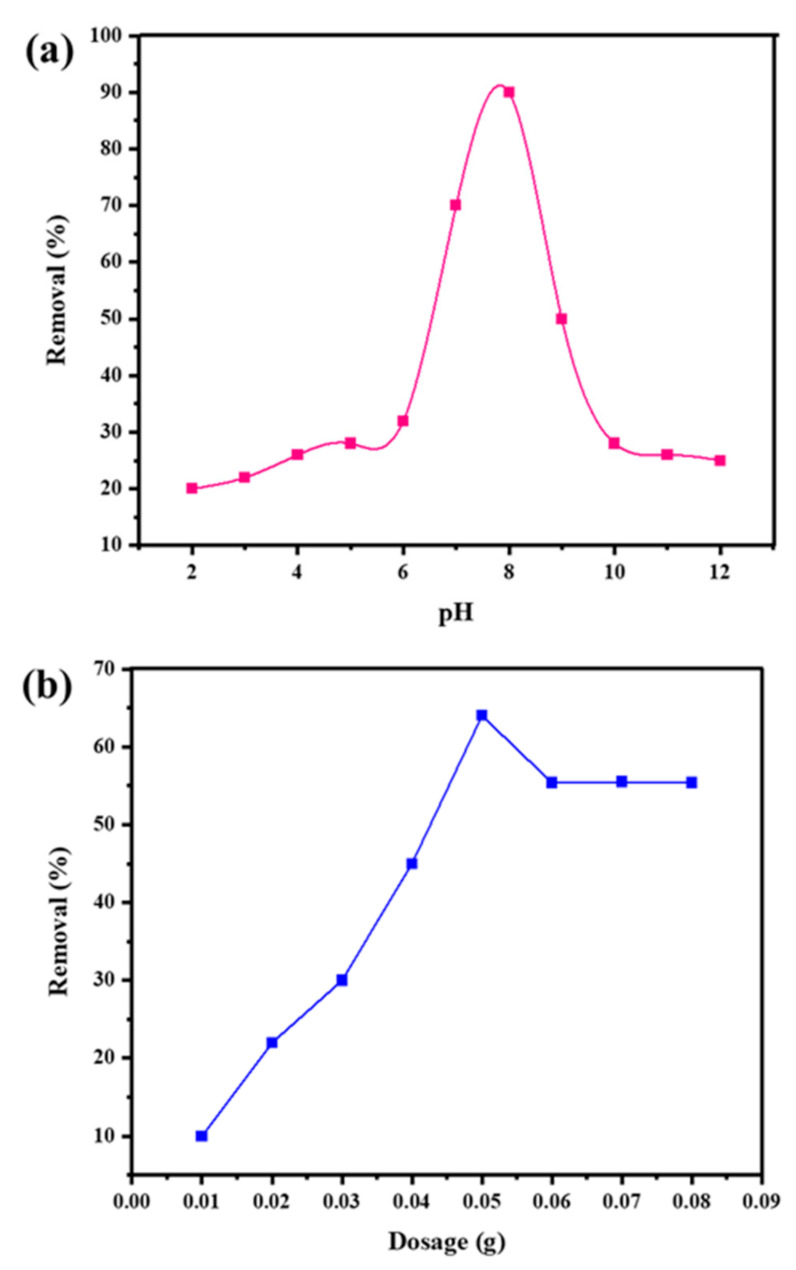
Effect of change in pH (a) and change in dosage (b) of Fr-MMWCNT-Fe_3_O_4_ on the removal of copper ions.

Since pH impacts the charge on the surface of copper ions and adsorbents, the pH variation affects the effectiveness of metal removal. According to the results, copper ion removal efficiency increases up to a pH of 8, but it starts to decrease after that point. The impact of pH on the adsorption of Cu^2+^ ions in solution is illustrated by a q_e_ vs. pH graph (Figure 6a). From Figure 6b, it is evident that increasing the dosage of the adsorbent material raises the adsorption proficiency up to 0.05 g; however, higher dosages slightly decrease efficiency. When more adsorbent was added, more adsorption sites became available, which may have initially resulted in higher adsorption, but as the dosage was increased, adsorbent aggregation caused the value of q_e_ to decrease. Thus, the observed optimum dosage of the composite was 0.05 g [48].

**Figure 7:**
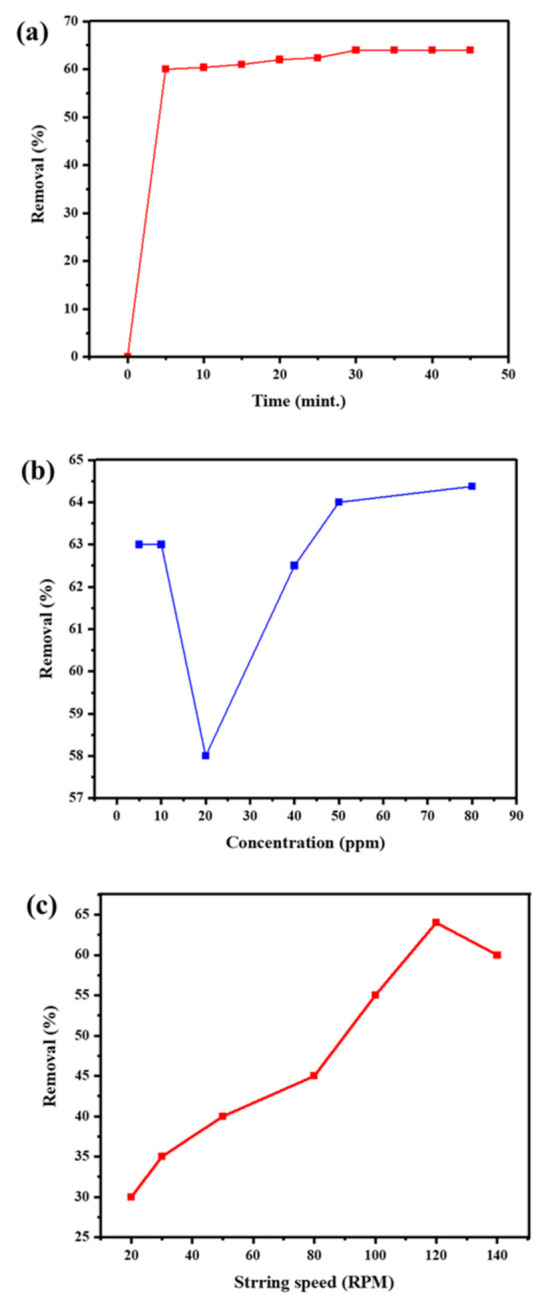
Effect of contact time (**a**), concentration of Cu^2+^ ions (**b**), and stirring time (**c**) on adsorption efficiency of Fr-MMWCNT-Fe3O4.

The effect of the contact time on the metal removal efficiency is exhibited in Figure 7a. As the contact time goes up, the removal rate goes up until 30 min, when it stays the same. Since, at the start, more empty sites are available; the Cu ion removal is fast. Nevertheless, as equilibrium is reached, it remains constant. Figure 7b explains the relationship between the adsorption efficiency of the Fr-MMWCNT-Fe_3_O_4_ composite and the initial concentration of Cu^2+^ ions. It is evident from Figure 7b that the removal efficiency also depends on the initial concentration of Cu^2+^ ions. The higher the initial concentrations of Cu^2+^ ions, the better the removal efficiency, and vice versa. The removal efficiency of Cu^2+^ ions shows a steady increase with an increase in concentration; however, a small dip is observed at 20 ppm, which could probably be due to an analysis error. Figure 7c shows that the stirring time has an impact on the removal efficiency of Cu^2+^ ions by the Fr-MMWCNT-Fe_3_O_4_ composite. The figure shows that as the stirring speed increases, the removal efficiency also increases. These results suggest that optimal conditions for the efficient removal of Cu^2+^ ions can be achieved by adjusting pH, dosage, contact time, initial concentration, and stirring speed.

### 2.3. Adsorption Isotherm Study

**Figure 8:**
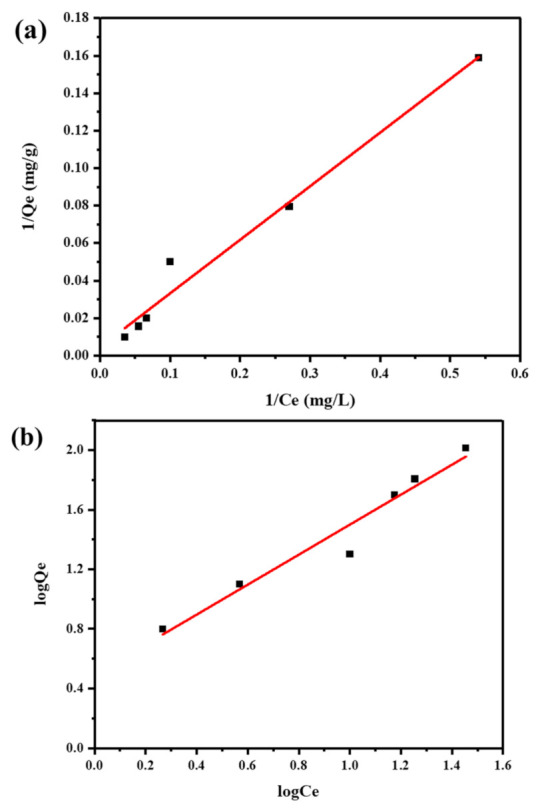
Adsorption isotherms of Cu^2+^ ions by Fr-MMWCNT-Fe_3_O_4_, the fitting plots of the Langmuir model (**a**), and Freundlich model (**b**).

Isotherms describe a solid-solution adsorption system’s adsorption behavior. The adsorption isotherm explains how adsorbate is distributed across the solid and liquid phases at equilibrium. The adsorption mechanism was investigated by utilizing the three adsorption isotherms. It was discovered that the Langmuir and Freundlich models better fit the equilibrium data than the Temkin model. These two models were better at predicting equilibrium data than the Temkin model. Langmuir yielded a maximum adsorption capacity (q_max_) of 250 mg/g and a Langmuir constant (K_L_) of 0.014 L/mg. The Freundlich model showed a high affinity for the adsorbate with a Freundlich constant (K_F_) of 3.119 (mg/g) × (L/mg)^(1/n)^ and a Freundlich exponent (n) of 0.994. The Temkin model had a lower R^2^ value and a weaker interaction [49]. Parameter results of the Langmuir, Freundlich, and Temkin models are mentioned in Table 1. The fact that Freundlich and Langmuir’s isotherms have a high correlation coefficient suggests that adsorbents have different kinds of active sites and that monolayer MB adsorption onto the Fr-MMWCNT-Fe_3_O_4_ occurs. Cu^2+^ ion adsorption was good. Overall, the Langmuir and Freundlich models provide a better fit for designing adsorption systems to remove the target adsorbate from the solution (Figure 8a,b).

### 2.4. Kinetics of Adsorption

**Figure 9:**
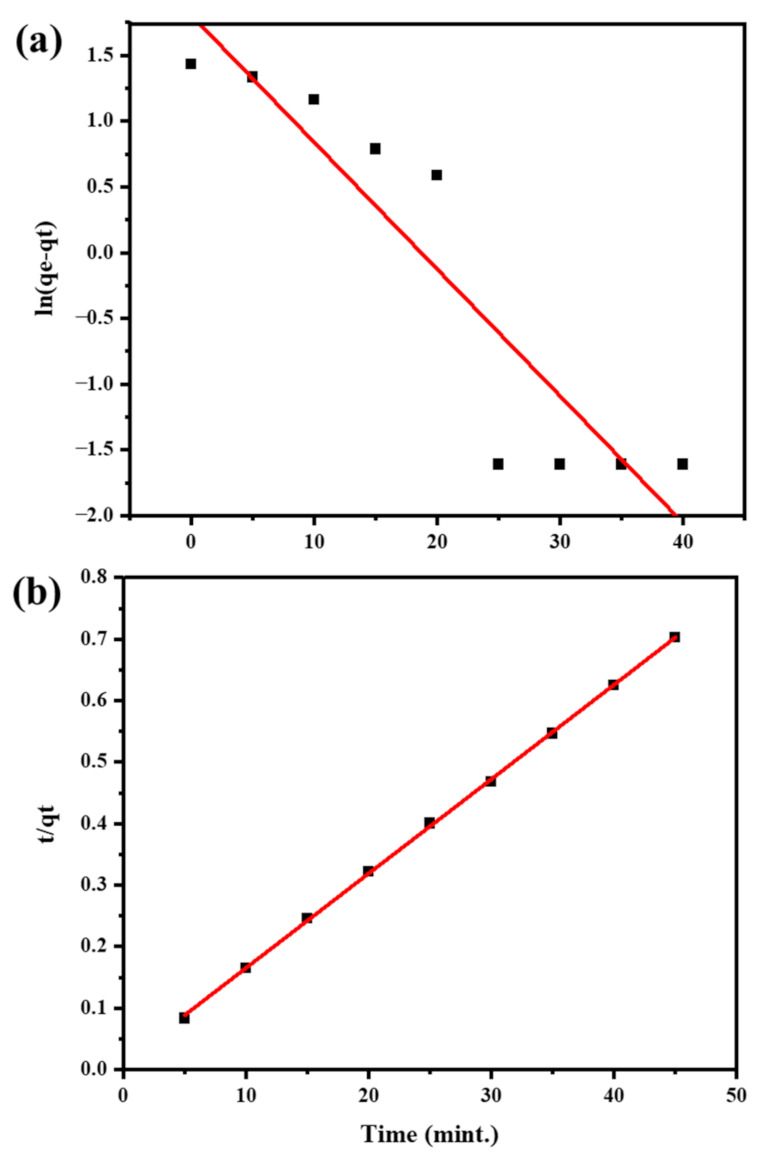
Adsorption kinetics of Cu^2+^ ions on the Fr-MMWCNT-Fe_3_O_4_ with kinetic modeling pseudo-first order (**a**) and pseudo-second order (**b**).

Adsorption kinetic models are essential for characterizing adsorption mechanisms due to their ability to predict equilibrium adsorption capacity and rate [50]. The PFO and PSO models are commonly used to describe the adsorption of a solute onto a solid adsorbent [51]. In contrast to the PSO model, which predicted a chemical reaction in adsorption, the PFO model predicted that physisorption was the primary catalyst for the reaction process. The given data provides information on the adsorption of Cu^2+^ ions on a nanocomposite at different temperatures. In the given data (Table 2), we can see that the values of k_1_ and R_2_ for the PFO model are reported for each temperature. The R_2_ values for the PFO model are comparatively low, which suggests that this model may not be the best fit for the adsorption system [52]. Therefore, it can be concluded that the rate of adsorption of Cu^2+^ ions on the nanocomposite is not well explained by the PFO model [53]. In the given data (Table 2), we can see that the values of k_2_ and R_2_ for the PSO model are reported for each temperature. The R_2_ values for the PSO model are relatively high, indicating that this model provides an excellent fit to the experimental data (Figure 9a,b) [54]. Therefore, it can be concluded that the rate of adsorption of Cu^2+^ ions on the nanocomposite is well described by the PSO model [55].

### 2.5. Thermodynamics of Adsorption

The thermodynamic parameters include the change in Gibbs free energy (ΔG°), the change in enthalpy (ΔH°), and the change in entropy (ΔS°) [56].

**Figure 10:**
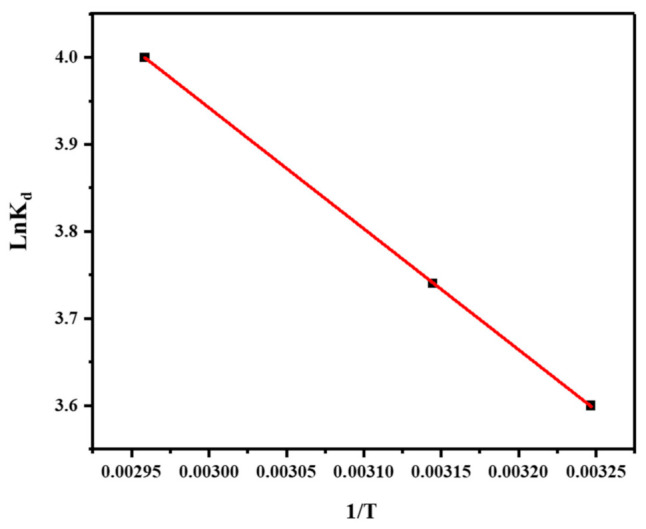
Thermodynamic modeling (Plot of lnK_d_ vs. 1/T).

The plot of LnK_d_ vs. 1/T is a representation of the thermodynamic model (shown in Figure 10 and Table 3). The data show that the ΔG° values at all temperatures are negative, indicating that the adsorption of Cu^2+^ ions onto the composite is a spontaneous process (Table 3) [57]. However, the values of ΔH° and ΔS° are only provided for the adsorption process at 308 K, with a ΔH° value of 11.62 kJ/mol and a ΔS° value of 67.42 J/mol.K [58]. These values indicate that the adsorption process is endothermic and leads to an increase in the disorder of the system [59]. 

### 2.6. Comparison of Adsorption Capacity of Fr-MMWCNT-Fe_3_O_4_ with Other Adsorbents

Table 4 presents a comparison of the removal efficiency of Cu^2+^ ions and the isotherm model for Fr-MMWCNT-Fe_3_O_4_ (current study) with those of other previous carbon-based adsorbents. Compared to the earlier reported data (Table 4), the maximum adsorption capacity of Fr-MMWCNT-Fe_3_O_4_ is significantly higher. This unequivocally establishes the efficacy of Fr-MMWCNT-Fe_3_O_4_ as adsorbents with regard to the removal of Cu^2+^ ions. The adsorption of Cu^2+^ ions on Fr-MMWCNT-Fe_3_O_4_ fits well with the Langmuir isotherm model, suggesting that a mono-layer adsorption process is taking place. This is also true for other types of adsorbents.

## 3. Materials and Methods

### 3.1. Materials

MWCNTs used in the study were procured from Adnano Technologies Private Limited, India. The MWCNTs had a purity of 99%, an outer diameter of 10–30 nm, an inner diameter of 5–10 nm, a length of over several micrometers, and a surface area of 110–350 m^2^/g. The frankincense gum used in the study was of the Hojari variety and was obtained from a local market. Analytical-grade ferric chloride and ferrous ammonium sulphate were purchased from EduChem in India. HCl and NaOH solutions were prepared at 0.1 M concentrations for pH adjustments and were obtained from SD Fine Chemical in India. Sulphuric acid (H_2_SO_4_, 98%) and nitric acid (HNO_3_, 71%) were purchased from Thomas Baker in India. 

### 3.2. Preparation of Fr-MMWCNT and Fr-MMWCNT-Fe_3_O_4_

To create the oxidized MMWCNT, 150 mL of a 1:1 mixture of concentrated HNO_3_ and H_2_SO_4_ was used to treat the MWCNT, which was then refluxed for 8 h. The resulting product was filtered, washed twice with distilled water (DW) until it reached a neutral pH, and dried. Fr-MMWCNT was prepared by stirring 0.4 g of Fr with 1.0 g of oxidized MWCNT at 50 °C for 8 h. The nanocomposite was collected via filtration, washed with DW, sonicated, and dried. The Fr-MMWCNT-Fe_3_O_4_ was produced by suspending 1.0 g of Fr-MWCNT in a 100 mL solution consisting of 0.845 g FeCl_3_·6H_2_O and 0.425 g FeSO_4_·7H_2_O, followed by the addition of 10 mL of an 8 mol L^−1^ NH_3_ solution to maintain the pH between 10 and 11. The mixture was stirred for 4 h at 60 °C under an N_2_ atmosphere and then washed with DW to reach a neutral pH. The subsequent composite was dried at approximately 80 °C. The magnetic composite, Fr-MMWCNT-Fe_3_O_4_, was characterized using various methods, including FT-IR, TGA, XRD, EDS, SEM, and HRTEM. 

### 3.3. Instrumentation

The FT-IR spectra of the Fr-MMWCNT-Fe_3_O_4_ composites were obtained using a Cary 630 FTIR instrument from Agilent Technologies (Stevens Creek, Santa Clara, CA, USA), within the range of 500 cm^−1^ to 4000 cm^−1^. XRD spectra were taken at room temperature using CuKα radiation (λ = 0.15406 nm, V = 30 kV, I = 30 mA) in the 2θ range of 4°–90°. SEM images were taken using two different instruments: VEGA 3 SBH from TESCAN Brno S.R.O. in the Czech Republic and SEM JEOL GSM 7600F, while EDS spectra were recorded on an SEM/EDS (at 5 kV voltage) instrument from EDAX Inc. (Pleasanton, CA, USA). High-resolution TEM images were captured at a resolution of 0.2 Å using a TEM-JEM-2100 plus instrument from JEOL (Tokyo, Japan). The absorbance of Cu^2+^ solutions was measured on an AA-7800 atomic absorption spectrophotometer (Tokyo, Japan). A 2100 pH meter from Pine Brook, (Parsippany, NJ 07054, USA) was used to measure pH. 

### 3.4. Adsorption Studies

To conduct the adsorption experiments, 0.05 g of composite was mixed with 100 mL of a copper sulphate solution at room temperature. The mixture was then magnetically stirred for 1 h, and subsequently centrifuged at 5000 RPM for 15 min. The AAS (AA-7800) was employed to measure the amount of copper adsorbed. The copper removal via adsorption was assessed by changing experimental conditions such as solution pH, contact time, stirring speed, solution concentration, and composite dose. The following equation was used to compute the % removal of copper:

% Removal of Copper = [(C_i_ − C_0_)/C_i_] × 100 (1)

where C_i_ and C_0_ are the initial and final concentrations of copper in mg/L.

The effect of pH on the adsorption of Cu^2+^ ions was studied using solutions with pH = 2–12 and measuring the absorbance at a constant temperature. The effect of stirring speed on copper removal was examined by changing it from 0 to 45 min. The effects of stirring speed on copper removal were studied by varying it from 20 to 140 RPM, while the effect of the number of nanoparticles used was studied by changing the dosage from 0.01 g to 0.08 g. Finally, the impact of the initial concentration of copper ions on the removal efficiency was examined by altering the concentration of the copper from 5 to 80 ppm in solution.

### 3.5. Adsorption Isotherm 

Langmuir, Freundlich, and Temkin are three commonly used models to describe the adsorption of Cu^2+^ ions onto a solid surface.

The Langmuir model is based on the assumption of monolayer adsorption, in which the surface contains a fixed number of active sites and each site can only adsorb one Cu ion [69]. According to this model (Equation (2)), the equilibrium adsorption capacity (q_e_) is related to the equilibrium concentration of Cu^2+^ ions in solution (C_e_) by the Langmuir isotherm equation [70]:

q_e_ = (q_max_ × K_L_ × C_e_)/(1 + K_L_ × C_e_) (2)

where q_max_ is the maximum adsorption capacity of the surface and K_L_ is the Langmuir constant, which is related to the affinity of the surface for Cu^2+^ ions.

Conversely, the Freundlich model proposes that adsorption occurs on a heterogeneous surface with various adsorption energies. According to this model, the q_e_ is related to the C_e_ of Cu^2+^ ions in solution by the Freundlich isotherm equation [71]:

q_e_ = K_F_ × C_e_^(1/n)^ (3)

The adsorption capacity of the surface is related to the Freundlich constant (K_F_), and the surface heterogeneity is related to the Freundlich linearity index (n).

The Temkin model proposes that the adsorption energy of Cu^2+^ ions on the surface decreases linearly with coverage, which means that the heat of adsorption declines logarithmically with surface coverage. According to this model, the q_e_ is related to the C_e_ of Cu^2+^ ions in solution by the Temkin isotherm equation [72]:

q_e_ = B ln(K_T_ C_e_) + B ln(A) (4)

where K_T_ is the Temkin constant, A is the heat of adsorption constant, and B is the universal gas constant. These models can be used to fit experimental data and determine the relevant parameters, such as q_max_, K_L_, K_F_, n, K_T_, and A, which provide insight into the adsorption behavior of Cu^2+^ ions on a surface.

### 3.6. Kinetics of Adsorption

To find the best-suited kinetic model, adsorption data was applied to pseudo-first-order (PFO) and pseudo-second-order (PSO) models to study the kinetics of the adsorption of Cu^2+^ ions onto adsorbent surfaces.

#### 3.6.1. Pseudo First Order

The PFO model is based on the supposition that the adsorption rate is directly proportional to the concentration of Cu^2+^ ions adhering to the adsorbent surface at any given time [73]. The mathematical equation for the PFO model is given as [74]:

log(q_e_ − q_t_) = logq_e_ − k_1_t/2.303

where q_e_ is the equilibrium adsorption capacity (mg/g), q_t_ is the amount of Cu^2+^ ions adsorbed on the adsorbent at time t (mg/g), k_1_ is the rate constant for the PFO model (min^−1^), and t is the time (min). By plotting log(q_e_ − q_t_) versus t, a linear graph can be obtained from which the rate constant can be calculated.

#### 3.6.2. Pseudo Second Order

The PSO model is based on the assumption that the adsorption rate is directly proportional to the square of the concentration of the Cu^2+^ ions remaining on the surface of the adsorbent at any given time. The mathematical equation for the PSO model is given as [75]:

t/q_t_ = 1/k_2_(q_e_^2^) + t/q_e_

where q_e_ is the equilibrium adsorption capacity (mg/g), q_t_ is the quantity of Cu^2+^ ions adsorbed on the adsorbent at time t (mg/g), k_2_ is the rate constant for the PSO model (g/mg min), and t is the time (min). By plotting t/q_t_ versus t, a linear graph can be obtained from which the rate constant and equilibrium adsorption capacity can be calculated. By comparing the correlation coefficients and other statistical parameters, the best-fit model can be selected for the Cu ion adsorption process.

### 3.7. The Thermodynamics of Adsorption

Thermodynamics of adsorption refers to the study of the energy changes that occur during the adsorption of a substance onto a surface. It helps in understanding the spontaneity, feasibility, and stability of the adsorption process.

The thermodynamic parameters used to describe adsorption are entropy change (ΔS°), Gibbs free energy change (ΔG°), and enthalpy change (ΔH°). The following equations are applied to calculate these parameters [56]:

ΔG° = −RTlnK                  

 ΔH° = −RT^2^(d lnK/d(1/T))

ΔS° = −R(d lnK/dT)          

where K and R are the equilibrium and gas constants, respectively, while T is the absolute temperature.

## 4. Conclusions

In this study, a novel composite of frankincense gum multi-walled carbon nanotubes with magnetic Fe_3_O_4_ nanoparticles (Fr-MMWCNT-Fe_3_O_4_) was prepared and characterized. The results indicated that the MWCNTs formed a composite with the Fr-gum and magnetite nanoparticles, with the latter randomly clustered in the gum material surrounding the CNTs. The composite was evaluated as an adsorbent for the removal of the emerging pollutant Cu^2+^ ions from an aqueous solution. The findings demonstrated that Fr-MMWCNT-Fe_3_O_4_ is a highly effective adsorbent for the remediation of Cu^2+^ ions, with a maximum adsorption capacity of 250 mg/g. The Langmuir model was found to be the most suitable for explaining the adsorption process, which was determined to be endothermic, spontaneous, and following a physisorption mechanism. This study suggests that more research is desirable to explore the potential of Fr-MMWCNT-Fe_3_O_4_ for adsorbing other pollutants, including pharmaceuticals, dyes, and organic substances.

## Figures and Tables

**Table 1:** Adsorption isotherm results for Cu^2+^ adsorption on Fr-MMWCNT-Fe_3_O_4_ at different temperatures.

Isotherm Models	Parameter Results	Study Temperatures (K)		
		308	318	338
Langmuir	q_m_ (mg/g)	250	240	239.5
	K_L_ (L/mg)	0.014	0.0132	0.011
	R^2^	0.972	0.9718	0.971
Freundlich	K_F_	3.119	3.0940	3.050
	n	0.994	0.9820	0.971
	R^2^	0.9488	0.939	0.940
Temkin	K_T_	0.3406	0.3550	0.3443
	B	67.750	67.815	67.912
	R^2^	0.6715	0.6823	0.6613

**Table 2:** Kinetic modeling results.

Study Temp (K)	q_e exp_ (mg/g)	Pseudo-First-Order Model			Pseudo-Second-Order Model		
		k_1_	q_e_	R^2^	k_2_	q_e_	R^2^
308	64.8	−0.0021	6.079	0.830	0.0197	65.14	0.999
318	65.0	−0.0018	7.022	0.811	0.0189	66.90	0.988
338	70.0	−0.0011	8.01	0.821	0.0190	67.60	0.984

**Table 3:** Thermodynamic study results.

Study Temp (K)	ΔG° (kJ/mol)	ΔH° (kJ/mol)	ΔS° (J/mol.K)
308	−3.28	11.62	67.42
318	−3.45		
338	−3.89		

**Table 4:** Maximum adsorption capacities (q_max_) and isotherm models of some reported carbon-based adsorbents for the removal of Cu^+2^.

Adsorbent	Isotherm Model	Adsorption Capacity (q_max_) mg/g	Reference
Fr-MMWCNT-Fe_3_O_4_	Langmuir	250	Present work
MMWCNTs	Langmuir	46.41	[12]
MWCNTs-COOH	Langmuir	10.45	[43]
CNTs-PDA-PP	Langmuir	26.40	[60]
NCNT-g-PMAA-2	Langmuir	89.00	[61]
c-MWCNTMCs	Langmuir	60.60	[62]
CNTs-CHO-CS	Langmuir	115.84	[63]
USSWCNT	Langmuir	142.86	[64]
NaOCl-modified CNTs	Langmuir	47.39	[65]
CNTs	Langmuir	28.49	[66]
SWCNT-COOH	Langmuir	77.00	[67]
CNT-PDA-CS	Langmuir	112.15	[68]

## Data Availability

Not applicable.

## Supplementary Materials

The following supporting information can be downloaded at: https://www.mdpi.com/article/10.3390/molecules28114494/s1, Figure S1. Diffraction peak spectra of Fr-MMWCNT. Figure S2. Diffraction peak spectra of Fr-MMWCNT-Fe_3_O_4_. References [76,77] are cited in the supplementary materials.

## Sample Availability

Samples of the compounds are available from the authors.
