# Supplementary material for: Enhancing Cu2+ Ion Removal: An Innovative Approach Utilizing Modified Frankincense Gum Combined with Multiwalled Carbon Tubes and Iron Oxide Nanoparticles as Adsorbent
Source: Molecules. 2023 Jun 1;28(11):4494. doi: 10.3390/molecules28114494 (PMC10254508; doi:10.3390/molecules28114494)
Supplement: Supplementary file 1 [file molecules-28-04494-s001.zip › molecules-2390365-supplementary.pdf]

Match! Phase Analysis Report

Sample: FRCNT-3

Sample Data

File name	FRCNT-3.txt
File path	C:/Users/musht/OneDrive/Desktop
Data collected	Aug 1, 2022 12:44:42
Data range	10.060º - 90.060º
Number of points	4001
Step size	0.020
Rietveld refinement converged	No
Alpha2 subtracted	Yes
Background subtr.	Yes
Data smoothed	No
2theta correction	0.06º
Radiation	X-rays
Wavelength	1.540598 Å

Matched Phases

Index	***Amount (%)***	***Name***	***Formula sum***
A	100.0	Carbon Graphite 2H	C
	99.1	*Unidentified peak area*	

A: Carbon Graphite 2H (100.0 %)

Formula sum	C
Entry number	96-101-1061
Figure-of-Merit (FoM)	0.745839
Total number of peaks	14
Peaks in range	14
Peaks matched	6
Intensity scale factor	0.99
Space group	P 63 m c
Crystal system	hexagonal
Unit cell	a= 2.4700 Å c= 6.7900 Å
I/Icor	2.58
Meas. density	2.160 g/cm³
Calc. density	2.221 g/cm³
Reference	Hassel O, "Ueber die Kristallstruktur des Graphits.", Zeitschrift fuer Physik **25**, 317-337 (1924)

Candidates

Name	***Formula***	***Entry No.***	***FoM***
Supercubane	C	96-901-2242	0.7733
Diamond	C	96-901-2237	0.7727
Carbon (Graphite 2H)	C	96-101-1061	0.7698
Carbon (Graphite 2H)	C	96-120-0018	0.7698
Graphite	C	96-901-2231	0.7604
Graphite	C	96-901-1578	0.7581
Carbon (Graphite 2H)	C	96-110-0004	0.7569
Carbon (Graphite 3R)	C	96-110-1022	0.7569
Carbon (Graphite 3R)	C	96-120-0019	0.7569
Graphite	C	96-901-2706	0.7569
Diamond	C	96-901-2236	0.7488
Graphite	C	96-900-0047	0.7473
Graphite	C	96-900-8570	0.7470
Graphite	C	96-901-2232	0.7422
Graphite	C	96-901-2234	0.7312
Graphite	C	96-901-2235	0.7254
Carbon (Lonsdaleite)	C	96-110-0005	0.7197
Carbon (Lonsdaleite)	C	96-110-1023	0.7197
Carbon (Lonsdaleite)	C	96-120-0020	0.7197
Graphite	C	96-901-2233	0.6838
Diamond	C	96-901-2238	0.6782
Carbon	C	96-901-2590	0.6697
Diamond	C	96-901-2239	0.6675
Diamond	C	96-901-2243	0.6476
Lonsdaleite	C	96-901-2471	0.6476
Carbon	C	96-901-2591	0.6416
Carbon	C	96-901-2595	0.6346
Carbon	C	96-901-2593	0.6164
Carbon	C	96-901-2594	0.6055

Search-Match

Settings

Reference database used	COD-Inorg REV173445 2016.01.04
Automatic zeropoint adaptation	Yes
Minimum figure-of-merit (FoM)	0.60
2theta window for peak corr.	0.30 deg.
Minimum rel. int. for peak corr.	1
Parameter/influence 2theta	0.50
Parameter/influence intensities	0.50
Parameter multiple/single phase(s)	0.50

Selection Criteria

Elements:

Elements that must be present:	C
Elements that must NOT be present:	All elements not mentioned above

Compound:

Formula sum:	C

Peak List

No.	***2theta [º]***	***d [Å]***	***I/I0***	***FWHM***	***Matched***
1	11.78	7.5078	90.97	0.1600	
2	11.96	7.3967	92.65	0.1600	
3	12.06	7.3355	97.15	0.1600	
4	12.15	7.2785	113.74	0.1600	
5	12.27	7.2086	109.83	0.1600	
6	12.36	7.1539	132.36	0.1600	
7	12.57	7.0372	129.75	0.1600	
8	12.69	6.9685	110.80	0.1600	
9	12.83	6.8930	121.29	0.1600	

10	13.04	6.7831	146.95	0.1600	
11	13.14	6.7304	156.75	0.1600	
12	13.33	6.6375	171.71	0.1600	
13	13.55	6.5285	150.53	0.1600	
14	13.76	6.4298	173.67	0.1600	
15	13.92	6.3551	158.44	0.1600	
16	14.04	6.3037	160.39	0.1600	
17	14.23	6.2182	188.07	0.1600	
18	14.41	6.1435	160.77	0.1600	
19	14.58	6.0689	151.32	0.1600	
20	14.79	5.9852	160.76	0.1600	
21	15.11	5.8598	181.15	0.1600	
22	15.35	5.7677	195.87	0.1600	
23	15.59	5.6779	191.58	0.1600	
24	15.76	5.6195	151.56	0.1600	
25	15.93	5.5585	167.18	0.1600	
26	16.07	5.5099	172.53	0.1600	
27	16.25	5.4486	169.59	0.1600	
28	16.43	5.3908	166.28	0.1600	
29	16.56	5.3496	184.31	0.1600	
30	16.69	5.3066	170.41	0.1600	
31	16.88	5.2491	181.43	0.1600	
32	17.08	5.1861	175.75	0.1600	
33	17.27	5.1311	176.84	0.1600	
34	17.51	5.0599	169.24	0.1600	
35	17.70	5.0060	178.26	0.1600	
36	17.90	4.9527	159.23	0.1600	
37	18.09	4.8985	172.50	0.1600	
38	18.26	4.8537	157.25	0.1600	
39	18.46	4.8017	169.46	0.1600	
40	18.73	4.7344	187.02	0.1600	
41	18.91	4.6889	166.86	0.1600	
42	19.08	4.6485	222.11	0.1600	
43	19.32	4.5909	197.13	0.1600	
44	19.46	4.5588	209.42	0.1600	
45	19.63	4.5197	222.04	0.1600	
46	19.89	4.4604	208.47	0.1600	
47	20.11	4.4129	214.60	0.1600	
48	20.25	4.3821	218.71	0.1600	
49	20.30	4.3714	242.06	0.1600	
50	20.50	4.3287	210.08	0.1600	
51	20.62	4.3030	217.89	0.1600	
52	20.79	4.2692	246.08	0.1600	
53	21.12	4.2039	245.51	0.1600	
54	21.29	4.1692	268.56	0.1600	
55	21.47	4.1358	270.92	0.1600	
56	21.70	4.0913	283.01	0.1600	
57	21.93	4.0493	306.72	0.1600	
58	22.03	4.0310	314.77	0.1600	
59	22.20	4.0018	316.45	0.1600	
60	22.40	3.9664	326.69	0.1600	
61	22.54	3.9407	321.09	0.1600	
62	22.60	3.9304	355.57	0.1600	
63	22.73	3.9089	356.57	0.1600	
64	22.93	3.8759	357.30	0.1600	
65	23.00	3.8643	372.37	0.1600	
66	23.18	3.8338	428.35	0.1600	
67	23.31	3.8138	398.71	0.1600	
68	23.42	3.7947	425.44	0.1600	
69	23.54	3.7761	430.90	0.1600	
70	23.75	3.7439	381.84	0.1600	
71	23.87	3.7244	437.78	0.1600	
72	24.04	3.6990	518.20	0.1600	
73	24.24	3.6688	222.76	0.1600	
74	24.29	3.6618	498.89	0.1600	
75	24.41	3.6437	568.77	0.1600	
76	24.60	3.6158	590.27	0.1600	
77	24.69	3.6024	619.32	0.1600	
78	24.80	3.5879	651.35	0.1600	
79	24.90	3.5731	637.23	0.1600	
80	25.10	3.5446	709.11	0.1600	
81	25.30	3.5170	703.58	0.1600	
82	25.38	3.5061	768.38	0.1600	
83	25.49	3.4918	798.01	0.1600	
84	25.56	3.4825	818.28	0.1600	
85	25.65	3.4703	858.26	0.1600	
86	25.76	3.4557	899.80	0.1600	
87	25.97	3.4282	971.86	0.1600	
88	26.12	3.4086	988.25	0.1600	
89	26.27	3.3900	963.08	0.1600	A
90	26.46	3.3657	1000.00	0.1600	
91	26.68	3.3391	887.67	0.1600	
92	26.80	3.3239	523.78	0.1600	
93	26.98	3.3023	800.93	0.1600	
94	27.06	3.2925	474.08	0.1600	
95	27.24	3.2707	741.39	0.1600	
96	27.43	3.2488	712.61	0.1600	
97	27.58	3.2316	434.15	0.1600	
98	27.79	3.2077	694.04	0.1600	
99	27.96	3.1885	343.49	0.1600	
100	28.14	3.1688	683.59	0.1600	
101	28.32	3.1483	630.91	0.1600	
102	28.48	3.1315	334.16	0.1600	
103	28.66	3.1118	618.05	0.1600	
104	28.82	3.0957	612.34	0.1600	
105	29.02	3.0745	636.19	0.1600	
106	29.20	3.0559	654.67	0.1600	
107	29.40	3.0358	652.68	0.1600	
108	29.60	3.0157	612.12	0.1600	
109	29.76	2.9998	576.05	0.1600	
110	29.93	2.9834	627.87	0.1600	
111	30.06	2.9709	610.89	0.1600	
112	30.18	2.9589	257.14	0.1600	
113	30.32	2.9459	569.37	0.1600	
114	30.45	2.9330	585.03	0.1600	
115	30.60	2.9192	259.41	0.1600	
116	30.72	2.9085	567.55	0.1600	
117	30.82	2.8992	531.27	0.1600	
118	30.92	2.8898	573.98	0.1600	
119	31.03	2.8798	509.09	0.1600	
120	31.23	2.8615	509.58	0.1600	
121	31.34	2.8520	538.30	0.1600	
122	31.47	2.8409	548.21	0.1600	

123	31.61	2.8285	485.77	0.1600	
124	31.74	2.8167	493.49	0.1600	
125	31.85	2.8077	494.86	0.1600	
126	32.09	2.7873	482.03	0.1600	
127	32.23	2.7749	528.67	0.1600	
128	32.38	2.7627	162.17	0.1600	
129	32.42	2.7593	458.91	0.1600	
130	32.56	2.7480	465.48	0.1600	
131	32.73	2.7341	573.57	0.1600	
132	32.87	2.7228	446.66	0.1600	
133	32.97	2.7146	488.60	0.1600	
134	33.08	2.7058	163.54	0.1600	
135	33.21	2.6953	431.34	0.1600	
136	33.40	2.6806	128.69	0.1600	
137	33.67	2.6595	482.21	0.1600	
138	33.78	2.6513	96.44	0.1600	
139	33.89	2.6431	482.82	0.1600	
140	34.03	2.6325	436.96	0.1600	
141	34.14	2.6242	131.45	0.1600	
142	34.32	2.6108	137.96	0.1600	
143	34.44	2.6023	367.00	0.1600	
144	34.56	2.5933	376.00	0.1600	
145	34.72	2.5819	405.17	0.1600	
146	34.89	2.5697	418.54	0.1600	
147	35.20	2.5476	417.42	0.1600	
148	35.36	2.5361	313.01	0.1600	
149	35.53	2.5249	442.64	0.1600	
150	35.72	2.5118	350.40	0.1600	
151	36.07	2.4882	420.96	0.1600	
152	36.32	2.4716	386.80	0.1600	
153	36.56	2.4555	348.60	0.1600	
154	36.73	2.4451	362.20	0.1600	
155	36.88	2.4353	339.39	0.1600	
156	37.00	2.4275	288.48	0.1600	
157	37.17	2.4169	340.10	0.1600	
158	37.38	2.4036	360.60	0.1600	
159	37.56	2.3925	360.71	0.1600	
160	37.80	2.3779	333.51	0.1600	
161	37.90	2.3717	330.63	0.1600	
162	38.10	2.3601	330.08	0.1600	
163	38.30	2.3481	414.41	0.1600	
164	38.51	2.3360	381.39	0.1600	
165	38.74	2.3226	327.10	0.1600	
166	38.98	2.3089	403.29	0.1600	
167	39.22	2.2953	391.87	0.1600	
168	39.42	2.2839	330.10	0.1600	
169	39.67	2.2701	354.57	0.1600	
170	39.87	2.2592	307.73	0.1600	
171	40.10	2.2468	91.51	0.1600	
172	40.35	2.2336	344.96	0.1600	
173	40.55	2.2232	309.08	0.1600	
174	40.75	2.2124	363.05	0.1600	
175	40.88	2.2057	333.72	0.1600	
176	41.15	2.1921	291.39	0.1600	
177	41.26	2.1861	319.91	0.1600	
178	41.47	2.1759	328.01	0.1600	
179	41.79	2.1598	309.90	0.1600	
180	41.96	2.1513	343.23	0.1600	
181	42.15	2.1421	337.84	0.1600	A
182	42.30	2.1348	291.92	0.1600	
183	42.51	2.1249	340.90	0.1600	
184	42.76	2.1130	108.93	0.1600	
185	43.00	2.1019	367.51	0.1600	
186	43.18	2.0934	328.34	0.1600	
187	43.29	2.0885	317.58	0.1600	
188	43.42	2.0823	309.29	0.1600	
189	43.58	2.0750	315.91	0.1600	
190	43.79	2.0655	337.57	0.1600	
191	44.02	2.0553	338.05	0.1600	
192	44.17	2.0489	292.94	0.1600	
193	44.42	2.0379	299.43	0.1600	A
194	44.68	2.0264	314.56	0.1600	
195	44.87	2.0185	265.19	0.1600	
196	45.04	2.0111	287.41	0.1600	
197	45.20	2.0042	205.12	0.1600	
198	45.54	1.9902	262.72	0.1600	
199	45.80	1.9796	185.43	0.1600	
200	45.91	1.9752	209.05	0.1600	
201	46.23	1.9623	179.32	0.1600	
202	46.57	1.9484	193.11	0.1600	
203	46.75	1.9413	154.97	0.1600	
204	46.90	1.9355	149.38	0.1600	
205	47.08	1.9286	197.72	0.1600	
206	47.32	1.9193	184.66	0.1600	
207	47.46	1.9141	152.11	0.1600	
208	47.62	1.9079	155.49	0.1600	
209	47.80	1.9012	142.64	0.1600	
210	47.96	1.8953	112.65	0.1600	
211	48.13	1.8891	133.69	0.1600	
212	48.38	1.8798	100.94	0.1600	
213	48.54	1.8740	116.34	0.1600	
214	48.89	1.8615	140.95	0.1600	
215	49.20	1.8504	144.81	0.1600	
216	49.44	1.8421	128.96	0.1600	
217	49.69	1.8334	139.19	0.1600	
218	49.90	1.8262	96.72	0.1600	
219	50.04	1.8213	99.90	0.1600	
220	50.31	1.8122	102.92	0.1600	
221	50.43	1.8082	93.94	0.1600	A
222	50.69	1.7994	103.39	0.1600	
223	50.90	1.7924	98.21	0.1600	
224	51.40	1.7762	103.08	0.1600	
225	52.32	1.7470	96.66	0.1600	
226	52.83	1.7315	89.76	0.1600	
227	52.95	1.7279	99.52	0.1600	
228	53.25	1.7187	88.57	0.1600	
229	53.62	1.7077	117.29	0.1600	
230	53.84	1.7014	96.50	0.1600	A
231	54.12	1.6932	95.78	0.1600	
232	54.40	1.6853	96.89	0.1600	
233	54.55	1.6808	88.28	0.1600	
234	54.66	1.6777	89.38	0.1600	
235	54.94	1.6700	117.98	0.1600	

236	55.80	1.6461	108.25	0.1600	
237	56.39	1.6303	96.97	0.1600	
238	56.71	1.6218	93.42	0.1600	
239	57.06	1.6129	111.82	0.1600	
240	57.54	1.6006	116.58	0.1600	
241	58.33	1.5806	103.35	0.1600	
242	58.44	1.5779	94.07	0.1600	
243	58.84	1.5681	108.07	0.1600	
244	59.33	1.5564	106.35	0.1600	A
245	59.67	1.5483	113.92	0.1600	
246	59.95	1.5419	87.52	0.1600	
247	60.31	1.5333	129.44	0.1600	
248	60.63	1.5261	100.84	0.1600	
249	60.94	1.5190	89.40	0.1600	
250	61.17	1.5139	88.42	0.1600	
251	61.70	1.5021	92.51	0.1600	
252	61.99	1.4958	103.56	0.1600	
253	62.40	1.4869	90.09	0.1600	
254	62.53	1.4841	108.21	0.1600	
255	64.47	1.4442	93.84	0.1600	
256	69.79	1.3466	93.80	0.1600	

Rietveld Refinement using FullProf

Calculation was not run or did not converge.

Integrated Profile Areas

Based on calculated profile

Profile area	***Counts***	***Amount***
Overall diffraction profile	199977	100.00%
Background radiation	31999	16.00%
Diffraction peaks	167977	84.00%
Peak area belonging to selected phases	1888	0.94%
Peak area of phase A (Carbon Graphite 2H)	*1888*	*0.94%*
Unidentified peak area	198088	99.06%

Peak Residuals

Peak data	***Counts***	***Amount***
Overall peak intensity	4491	100.00%
Peak intensity belonging to selected phases	72	1.60%
Unidentified peak intensity	4419	98.40%

Diffraction Pattern Graphics

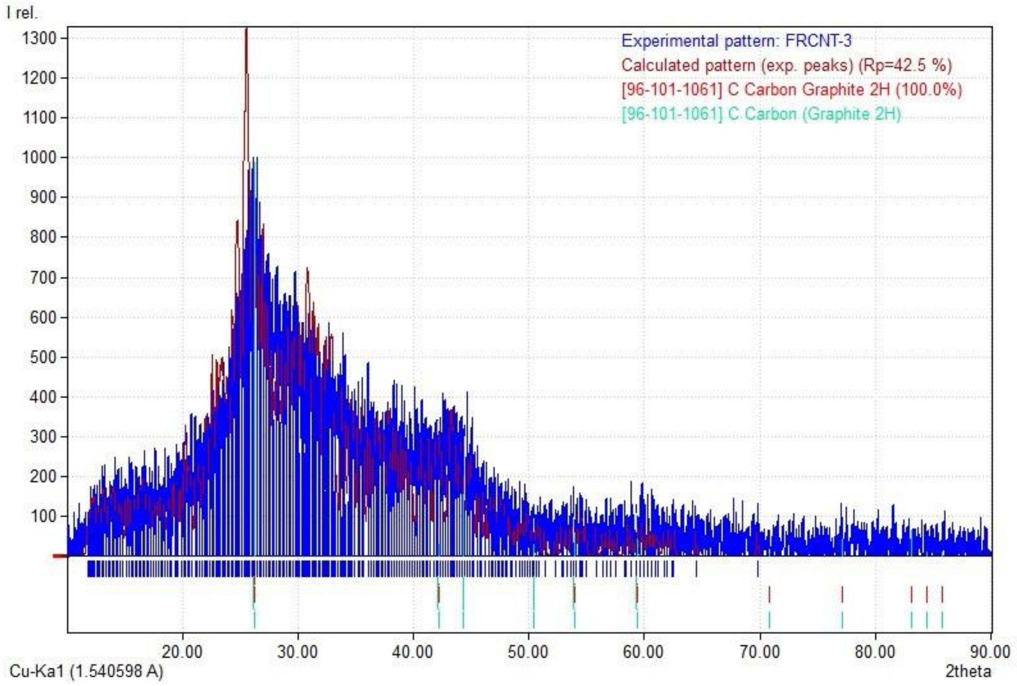

Figure S1. Diffraction peak spectra of Fr-MMWCNT.

Match! Copyright © 2003-2016 CRYSTAL IMPACT, Bonn, Germany

Match! Phase Analysis Report

Sample: Fr-MMWCNT-Fe3O4

Sample Data

File name	Fr-MMWCNT-Fe3O4.txt
File path	C:/Users/musht/OneDrive/Desktop
Data collected	May 9, 2023 10:19:13
Data range	24.930º - 89.930º
Number of points	3251
Step size	0.020
Rietveld refinement converged	No
Alpha2 subtracted	Yes
Background subtr.	Yes
Data smoothed	No
2theta correction	-0.07º
Radiation	X-rays
Wavelength	1.540598 Å

Matched Phases

Index	*Amount (%)*	*Name*	*Formula sum*
A	100.0	Magnetite	Fe3 O4
	49.9	*Unidentified peak area*	

A: Magnetite (100.0 %)

Formula sum	Fe3 O4
Entry number	96-900-2321
Figure-of-Merit (FoM)	0.828991
Total number of peaks	68
Peaks in range	34
Peaks matched	27
Intensity scale factor	0.96
Space group	F d -3 m
Crystal system	cubic
Unit cell	a= 8.3122 Å
I/Icor	6.03
Calc. density	5.355 g/cm³
Reference	Haavik C., Stolen S., Fjellvag H., Hanfland M., Hausermann D., "Equation of state of magnetite and its high-pressure modification: Thermodynamics of the Fe-O system at high pressure Sample at P = 6.6 GPa", American Mineralogist **85**, 514-523 (2000)

Candidates

Name	*Formula*	*Entry No.*	*FoM*
Fe2 O3	Fe2 O3	96-152-8613	0.8419
Magnetite	Fe3 O4	96-900-2320	0.8348
Magnetite	Fe3 O4	96-900-5841	0.8328
Magnetite	Fe3 O4	96-900-5814	0.8322
Magnetite	Fe3 O4	96-900-5840	0.8233
Magnetite	Fe3 O4	96-900-5839	0.8216
Magnetite	Fe3 O4	96-900-2324	0.8209
	Fe3 O4	96-210-1927	0.8207
Magnetite	Fe3 O4	96-900-2319	0.8207
Magnetite	Fe3 O4	96-900-5813	0.8199
Magnetite	Fe3 O4	96-900-2321	0.8194
Magnetite	Fe3 O4	96-900-5817	0.8129
Magnetite	Fe3 O4	96-900-2323	0.8115
Magnetite	Fe3 O4	96-901-0942	0.8100
Magnetite	Fe3 O4	96-900-2322	0.8080
Magnetite	Fe3 O4	96-900-9769	0.8072
Iron diiron(III) oxide (Magnetite)	Fe3 O4	96-101-1033	0.8067
(Fe2 O3)10.6667	Fe21.3334 O32.0001	96-152-8612	0.8063
Maghemite	Fe3 O4	96-900-6319	0.8062
	Fe3 O4	96-210-1536	0.8060
Magnetite	Fe3 O4	96-900-2674	0.8060
Magnetite	Fe3 O4	96-900-5838	0.8054
Magnetite	Fe3 O4	96-900-5843	0.8053
Magnetite	Fe3 O4	96-900-2675	0.8052
Magnetite	Fe3 O4	96-900-9770	0.8049
Magnetite	Fe3 O4	96-900-2327	0.8039
Maghemite	Fe2 O3	96-901-2693	0.8026
Magnetite	Fe3 O4	96-900-5842	0.8023
Magnetite	Fe3 O4	96-900-6248	0.8021
Iron diiron(III) oxide	Fe3 O4	96-101-0370	0.8015
Magnetite	Fe3 O4	96-901-0941	0.7998
Magnetite	Fe3 O4	96-900-5816	0.7995
Magnetite	Fe3 O4	96-900-2318	0.7991
Maghemite	Fe2 O3	96-900-6317	0.7987
Iron diiron(III) oxide (Magnetite)	Fe3 O4	96-101-1085	0.7980
iron oxide	Fe3 O4	96-151-3305	0.7956
Magnetite	Fe3 O4	96-900-2328	0.7942
iron oxide	Fe3 O4	96-151-3302	0.7938
Fe3 O4	Fe3 O4	96-152-6956	0.7931
Magnetite	Fe3 O4	96-900-6253	0.7928
Magnetite	Fe3 O4	96-900-7707	0.7928
Magnetite	Fe3 O4	96-900-6185	0.7927
Magnetite	Fe3 O4	96-900-6190	0.7927
Magnetite	Fe3 O4	96-900-6195	0.7927
Magnetite	Fe3 O4	96-900-6243	0.7927
Magnetite	Fe3 O4	96-900-6266	0.7927
Magnetite	Fe3 O4	96-901-3530	0.7927
Magnetite	Fe3 O4	96-900-6200	0.7923
Magnetite	Fe3 O4	96-900-6921	0.7923
Magnetite	Fe3 O4	96-901-0940	0.7923
Magnetite	Fe3 O4	96-900-7645	0.7922
Magnetite	Fe3 O4	96-900-5837	0.7920

and 38 others...

Search-Match

Settings

Reference database used	COD-Inorg REV173445 2016.01.04
Automatic zeropoint adaptation	Yes
Minimum figure-of-merit (FoM)	0.60
2theta window for peak corr.	0.30 deg.
Minimum rel. int. for peak corr.	1
Parameter/influence 2theta	0.50
Parameter/influence intensities	0.50

Parameter multiple/single phase(s) 0.50

Selection Criteria

Elements:

Elements that must be present:	O, Fe
Elements that must NOT be present:	All elements not mentioned above

Peak List

No.	*2theta [º]*	*d [Å]*	*I/I0*	*FWHM*	*Matched*
1	25.46	3.4953	62.52	0.9200	
2	25.70	3.4639	122.16	0.9200	
3	25.79	3.4517	143.89	0.9200	
4	25.89	3.4381	185.76	0.9200	
5	26.05	3.4178	193.95	0.9200	
6	26.19	3.3997	218.91	0.9200	
7	26.36	3.3779	207.02	0.9200	
8	26.53	3.3571	173.54	0.9200	
9	26.71	3.3344	202.66	0.9200	
10	26.91	3.3105	173.14	0.9200	
11	27.08	3.2904	155.46	0.9200	
12	27.21	3.2747	130.22	0.9200	
13	27.37	3.2563	93.20	0.9200	
14	27.54	3.2364	111.25	0.9200	
15	27.75	3.2122	84.58	0.9200	
16	27.87	3.1992	104.25	0.9200	
17	27.99	3.1852	83.78	0.9200	
18	28.33	3.1477	50.74	0.9200	
19	28.59	3.1202	42.15	0.9200	
20	28.76	3.1012	42.05	0.9200	
21	29.51	3.0245	47.61	0.9200	
22	29.65	3.0104	88.65	0.9200	
23	29.85	2.9905	109.84	0.9200	
24	30.01	2.9752	153.87	0.9200	
25	30.17	2.9598	178.23	0.9200	
26	30.29	2.9484	259.10	0.9200	
27	30.37	2.9408	287.21	0.9200	A
28	30.57	2.9224	342.48	0.9200	A
29	30.73	2.9071	298.38	0.9200	
30	31.07	2.8761	158.38	0.9200	
31	31.21	2.8635	115.24	0.9200	
32	31.39	2.8476	106.71	0.9200	
33	31.51	2.8369	82.32	0.9200	
34	31.63	2.8265	61.21	0.9200	
35	32.11	2.7853	57.13	0.9200	
36	32.24	2.7744	92.51	0.9200	
37	32.45	2.7572	100.92	0.9200	
38	32.61	2.7437	93.83	0.9200	
39	32.75	2.7323	79.71	0.9200	
40	32.88	2.7214	70.81	0.9200	
41	33.02	2.7109	54.25	0.9200	
42	33.64	2.6623	48.52	0.9200	
43	33.83	2.6479	102.92	0.9200	
44	34.03	2.6328	143.03	0.9200	
45	34.18	2.6209	134.16	0.9200	
46	34.35	2.6089	96.38	0.9200	
47	34.47	2.5998	89.91	0.9200	
48	34.59	2.5911	74.22	0.9200	
49	34.93	2.5666	76.42	0.9200	
50	35.11	2.5539	151.45	0.9200	
51	35.29	2.5412	330.70	0.9200	
52	35.37	2.5357	414.81	0.9200	
53	35.49	2.5274	617.30	0.9200	
54	35.59	2.5205	740.71	0.9200	
55	35.67	2.5150	830.74	0.9200	
56	35.73	2.5110	904.32	0.9200	
57	35.77	2.5082	941.18	0.9200	A
58	35.91	2.4985	1000.00	0.9200	A
59	36.05	2.4894	921.42	0.9200	
60	36.09	2.4867	873.77	0.9200	
61	36.29	2.4735	591.88	0.9200	
62	36.37	2.4682	461.26	0.9200	
63	36.43	2.4643	367.38	0.9200	
64	36.57	2.4552	200.18	0.9200	
65	36.69	2.4474	146.46	0.9200	
66	36.87	2.4359	72.87	0.9200	
67	37.12	2.4204	105.95	0.9200	
68	37.23	2.4131	112.05	0.9200	
69	37.45	2.3996	140.68	0.9200	A
70	37.60	2.3905	129.91	0.9200	A
71	37.79	2.3787	75.24	0.9200	
72	37.99	2.3667	61.46	0.9200	
73	38.19	2.3549	41.47	0.9200	
74	38.33	2.3464	49.78	0.9200	
75	39.24	2.2943	51.30	0.9200	
76	39.78	2.2641	45.49	0.9200	
77	40.01	2.2517	65.98	0.9200	
78	40.13	2.2452	40.93	0.9200	
79	40.86	2.2069	76.60	0.9200	
80	41.07	2.1958	74.22	0.9200	
81	41.25	2.1870	98.76	0.9200	
82	42.71	2.1154	88.41	0.9200	
83	42.93	2.1050	135.02	0.9200	
84	43.05	2.0994	162.51	0.9200	
85	43.23	2.0911	199.20	0.9200	
86	43.43	2.0821	217.12	0.9200	
87	43.59	2.0747	178.10	0.9200	A
88	43.71	2.0693	176.53	0.9200	
89	43.92	2.0598	137.61	0.9200	
90	44.15	2.0495	89.01	0.9200	
91	44.43	2.0374	50.17	0.9200	
92	44.71	2.0255	65.75	0.9200	
93	44.85	2.0193	47.00	0.9200	
94	44.96	2.0147	69.93	0.9200	
95	45.11	2.0082	61.05	0.9200	
96	45.27	2.0013	67.04	0.9200	
97	45.54	1.9904	83.97	0.9200	
98	45.79	1.9800	91.68	0.9200	
99	45.93	1.9743	61.08	0.9200	
100	46.04	1.9698	77.66	0.9200	
101	46.28	1.9601	102.13	0.9200	
102	46.43	1.9542	78.21	0.9200	
103	46.74	1.9419	97.01	0.9200	

104	47.47	1.9138	49.15	0.9200	
105	47.62	1.9080	68.99	0.9200	A
106	47.86	1.8991	53.68	0.9200	A
107	48.03	1.8927	53.16	0.9200	
108	48.21	1.8861	43.24	0.9200	
109	49.05	1.8557	87.95	0.9200	
110	49.27	1.8480	95.39	0.9200	
111	49.55	1.8382	74.54	0.9200	
112	49.73	1.8319	57.26	0.9200	
113	49.92	1.8253	92.15	0.9200	
114	50.11	1.8189	76.01	0.9200	
115	50.43	1.8082	62.88	0.9200	
116	50.63	1.8015	56.28	0.9200	
117	50.79	1.7962	67.00	0.9200	
118	50.95	1.7909	57.01	0.9200	
119	51.63	1.7689	84.99	0.9200	
120	51.92	1.7596	103.94	0.9200	
121	52.03	1.7562	63.18	0.9200	
122	52.31	1.7475	56.01	0.9200	
123	52.53	1.7407	85.66	0.9200	
124	52.69	1.7358	89.69	0.9200	
125	52.87	1.7303	68.68	0.9200	
126	52.97	1.7273	61.71	0.9200	
127	53.29	1.7176	78.63	0.9200	
128	53.47	1.7123	77.07	0.9200	
129	53.59	1.7087	70.55	0.9200	
130	53.76	1.7038	116.32	0.9200	
131	53.87	1.7005	90.21	0.9200	
132	53.99	1.6970	110.26	0.9200	A
133	54.15	1.6924	117.61	0.9200	A
134	54.27	1.6889	129.40	0.9200	
135	54.45	1.6838	137.73	0.9200	
136	54.73	1.6757	127.46	0.9200	
137	54.95	1.6696	50.00	0.9200	
138	55.13	1.6646	42.81	0.9200	
139	55.47	1.6552	106.93	0.9200	
140	55.67	1.6498	81.35	0.9200	
141	56.39	1.6304	66.01	0.9200	
142	56.50	1.6275	89.07	0.9200	
143	56.65	1.6235	108.91	0.9200	
144	56.75	1.6209	192.03	0.9200	
145	56.89	1.6172	212.67	0.9200	
146	57.01	1.6141	213.38	0.9200	
147	57.13	1.6110	282.01	0.9200	
148	57.27	1.6073	302.92	0.9200	
149	57.47	1.6023	342.97	0.9200	
150	57.61	1.5987	368.73	0.9200	A
151	57.67	1.5972	356.58	0.9200	A
152	57.79	1.5941	264.78	0.9200	
153	57.85	1.5926	239.57	0.9200	
154	58.03	1.5881	157.85	0.9200	
155	58.15	1.5851	102.26	0.9200	
156	58.42	1.5784	75.08	0.9200	
157	58.74	1.5707	51.14	0.9200	
158	59.84	1.5444	70.08	0.9200	
159	60.15	1.5370	70.77	0.9200	
160	60.41	1.5311	71.37	0.9200	
161	60.58	1.5271	65.27	0.9200	
162	60.77	1.5229	52.05	0.9200	
163	60.88	1.5204	52.60	0.9200	
164	62.03	1.4950	51.07	0.9200	
165	62.25	1.4902	86.84	0.9200	
166	62.44	1.4861	165.05	0.9200	
167	62.61	1.4825	190.64	0.9200	
168	62.73	1.4800	288.99	0.9200	
169	62.89	1.4765	403.06	0.9200	
170	63.10	1.4722	454.48	0.9200	
171	63.27	1.4686	418.55	0.9200	A
172	63.39	1.4661	327.72	0.9200	A
173	63.53	1.4632	252.71	0.9200	
174	63.63	1.4612	195.47	0.9200	
175	63.73	1.4591	146.93	0.9200	
176	63.85	1.4567	129.32	0.9200	
177	64.05	1.4526	126.04	0.9200	
178	64.21	1.4494	76.39	0.9200	
179	64.37	1.4462	83.54	0.9200	
180	64.57	1.4422	66.73	0.9200	
181	64.78	1.4380	44.20	0.9200	
182	65.63	1.4214	73.33	0.9200	
183	65.81	1.4180	43.11	0.9200	
184	65.93	1.4157	42.10	0.9200	
185	66.07	1.4130	50.55	0.9200	
186	66.29	1.4088	48.79	0.9200	
187	66.50	1.4049	59.42	0.9200	A
188	66.73	1.4006	57.10	0.9200	A
189	66.83	1.3988	54.17	0.9200	
190	67.08	1.3941	50.00	0.9200	
191	67.26	1.3908	64.14	0.9200	
192	67.45	1.3873	89.30	0.9200	
193	67.63	1.3842	67.42	0.9200	
194	67.79	1.3813	93.23	0.9200	
195	68.75	1.3644	40.79	0.9200	
196	68.91	1.3615	54.16	0.9200	
197	69.08	1.3587	86.17	0.9200	
198	69.29	1.3550	69.58	0.9200	
199	69.46	1.3521	87.07	0.9200	
200	69.61	1.3495	85.77	0.9200	
201	69.85	1.3455	65.78	0.9200	
202	69.99	1.3431	50.14	0.9200	
203	70.44	1.3357	58.14	0.9200	
204	70.69	1.3315	44.22	0.9200	
205	70.83	1.3293	90.05	0.9200	
206	71.29	1.3218	42.23	0.9200	
207	71.57	1.3173	68.25	0.9200	
208	71.83	1.3132	79.95	0.9200	A
209	71.99	1.3107	84.66	0.9200	A
210	72.34	1.3052	52.29	0.9200	
211	72.79	1.2982	63.87	0.9200	
212	72.95	1.2958	45.20	0.9200	
213	73.12	1.2932	63.53	0.9200	
214	73.29	1.2906	56.91	0.9200	
215	73.43	1.2885	64.72	0.9200	
216	73.65	1.2851	84.85	0.9200	

217	73.79	1.2831	70.16	0.9200	
218	73.95	1.2807	99.14	0.9200	
219	74.06	1.2791	112.22	0.9200	
220	74.19	1.2772	98.81	0.9200	
221	74.33	1.2751	112.36	0.9200	
222	74.44	1.2735	134.13	0.9200	
223	75.21	1.2623	80.47	0.9200	A
224	75.37	1.2601	78.51	0.9200	
225	75.55	1.2575	82.62	0.9200	
226	75.89	1.2527	43.75	0.9200	A
227	76.05	1.2505	95.20	0.9200	A
228	76.15	1.2491	58.34	0.9200	
229	76.39	1.2457	53.57	0.9200	
230	77.20	1.2347	64.07	0.9200	
231	77.39	1.2321	91.43	0.9200	
232	77.53	1.2303	141.31	0.9200	
233	77.97	1.2244	63.13	0.9200	
234	78.07	1.2231	44.14	0.9200	
235	78.41	1.2186	59.19	0.9200	
236	78.54	1.2170	113.06	0.9200	
237	78.65	1.2155	71.00	0.9200	
238	78.89	1.2124	96.58	0.9200	
239	79.38	1.2061	51.08	0.9200	
240	79.73	1.2018	57.65	0.9200	
241	79.87	1.2000	57.55	0.9200	A
242	80.09	1.1973	109.43	0.9200	A
243	80.23	1.1955	116.86	0.9200	
244	80.35	1.1940	71.56	0.9200	
245	80.54	1.1917	93.05	0.9200	
246	81.29	1.1826	57.17	0.9200	
247	81.69	1.1778	64.33	0.9200	
248	81.80	1.1765	137.11	0.9200	
249	82.09	1.1731	73.43	0.9200	
250	82.19	1.1719	132.23	0.9200	
251	82.47	1.1686	69.38	0.9200	
252	82.66	1.1665	147.48	0.9200	
253	82.77	1.1652	106.54	0.9200	A
254	82.99	1.1626	113.99	0.9200	
255	83.15	1.1608	68.58	0.9200	A
256	83.37	1.1583	61.90	0.9200	
257	83.48	1.1571	100.35	0.9200	
258	83.67	1.1549	98.82	0.9200	
259	83.86	1.1528	163.46	0.9200	
260	84.03	1.1509	128.54	0.9200	
261	84.15	1.1496	118.94	0.9200	
262	84.29	1.1480	110.39	0.9200	
263	84.41	1.1466	95.60	0.9200	
264	84.61	1.1444	112.70	0.9200	
265	84.75	1.1429	133.89	0.9200	
266	84.87	1.1416	129.07	0.9200	
267	84.99	1.1403	148.06	0.9200	
268	85.16	1.1385	211.05	0.9200	
269	85.27	1.1373	183.93	0.9200	
270	85.47	1.1351	142.94	0.9200	
271	85.57	1.1340	137.33	0.9200	
272	85.81	1.1315	91.37	0.9200	
273	86.08	1.1286	97.45	0.9200	
274	86.21	1.1273	57.93	0.9200	
275	89.77	1.0916	104.24	0.9200	

Rietveld Refinement using FullProf

Calculation was not run or did not converge.

Integrated Profile Areas

Based on calculated profile

Profile area	***Counts***	***Amount***
Overall diffraction profile	22102	100.00%
Background radiation	102	0.46%
Diffraction peaks	22000	99.54%
Peak area belonging to selected phases	11066	50.06%
Peak area of phase A (Magnetite)	*11066*	*50.06%*
Unidentified peak area	11037	49.94%

Peak Residuals

Peak data	***Counts***	***Amount***
Overall peak intensity	4078	100.00%
Peak intensity belonging to selected phases	377	9.25%
Unidentified peak intensity	3701	90.75%

Diffraction Pattern Graphics

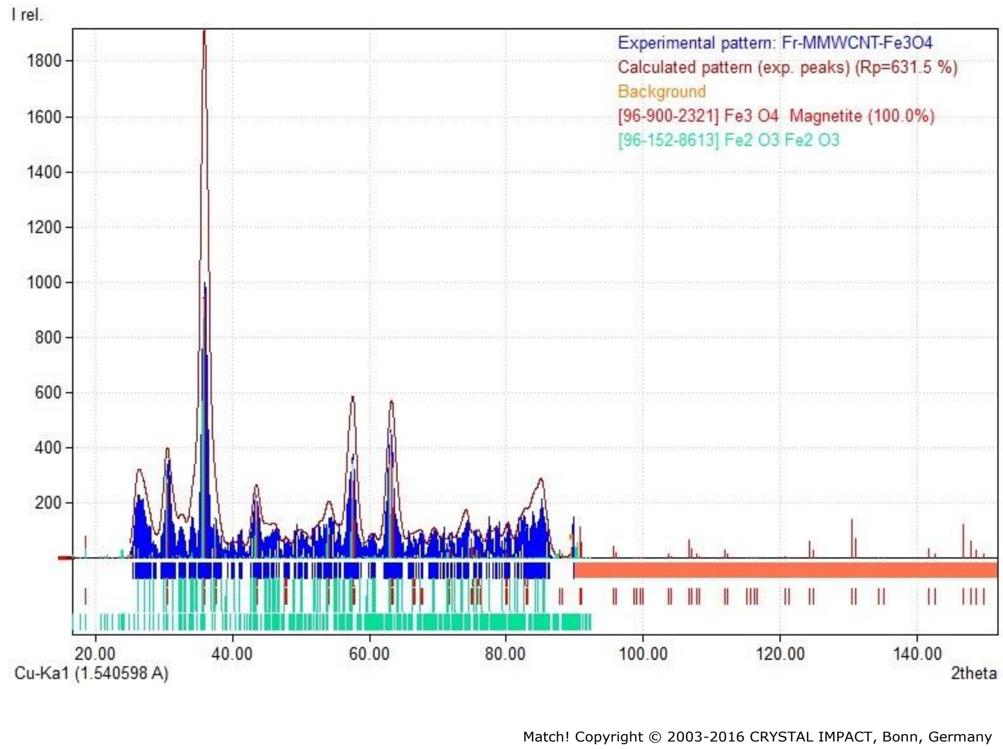

Figure S2. Diffraction peak spectra of Fr-MMWCNT-Fe_3O_4.
